# A Case of Rubella Caused by Rubella Vaccination

**DOI:** 10.3390/vaccines9091040

**Published:** 2021-09-18

**Authors:** Momoka Kamada, Tsuneaki Kenzaka

**Affiliations:** 1 Department of Internal Medicine, Hyogo Prefectural Tamba Medical Center, 2002-7 Iso, Hikami-cho, Tamba 669-3495, Japan; m08018mo@jichi.ac.jp; 2 Division of Community Medicine and Career Development, Kobe University Graduate School of Medicine, 2-1-5 Arata-cho, Hyogo-ku, Kobe 652-0032, Japan

**Keywords:** rubella, vaccine, adverse reaction, genotyping, vaccine strain, measles-rubella, rash, case report

## Abstract

We present an extremely rare case of rubella that developed after rubella vaccine administration. A 54-year-old man complained of back and neck pain for some days. He presented with generalized rash and arthralgia that had persisted for two days before his visit. His vital signs were normal, and arthralgia had disappeared during an examination, but lymphadenopathy in the left posterior neck and pink papules were observed throughout the body. He had received his first Rubella vaccination 17 days before this visit and had attended a crowded festival. Owing to the rubella epidemic in that prefecture, we performed a rubella antibody test and polymerase chain reaction assay using blood, urine, and pharyngeal swab specimens. Rubella IgG and IgM antibody titers were 3 and 1.48, respectively. The pharyngeal swab yielded positive results for the 1a vaccine strain. Therefore, he was diagnosed with rubella due to rubella vaccination. His symptoms improved eventually. His clinical course was uncomplicated. Symptoms resolved within one week without specific treatment. The vaccine rubella strain is not as highly infectious as wild-type rubella strains. If rubella symptoms appear after vaccination, it must be investigated whether these are vaccine-specific adverse reactions, wild-strain rubella onset, or other eruptive viral infections.

## 1. Introduction

Rubella is a viral disease characterized by fever, rash, and lymphadenopathy [1]. After an incubation period of 14–21 days, the three characteristic features appear (especially in the posterior pinna, occipital region, and neck area), with fever being present in approximately half of the rubella patients [1]. Furthermore, subclinical infections are present in approximately 15% of cases, and it is difficult to clinically diagnose cases wherein patients lack any of the three signs. Moreover, it is necessary to distinguish it from other fever–rash diseases and drug eruptions. Therefore, a laboratory diagnosis is required for a definitive diagnosis [2]. Rubella is diagnosed based on the following factors: (1) Isolation and identification of the virus using throat swab, blood, cerebrospinal fluid, and urine specimens obtained during the acute phase (a few days after the appearance of the rash), (2) detection of viral genes via the polymerase chain reaction (PCR) test using throat swab, blood, cerebrospinal fluid, and urine obtained during the acute phase, (3) detection of rubella IgM in the acute phase blood specimen, and (4) presence of either seroconversion or significantly elevated antibody titers in paired blood specimens in the acute phase and convalescent phase (approximately two weeks after onset) [2]. There is no specific treatment for rubella, and only symptomatic treatment is available.

Rubella also results in congenital infections. If a pregnant woman is infected with the rubella virus during the first 20 gestational weeks, the infection can spread to the fetus, causing congenital rubella syndrome, which includes congenital anomalies, such as heart disease, deafness, cataract, and retinitis pigmentosa. Furthermore, progressive rubella total encephalitis, diabetes mellitus, and psychomotor developmental delay have also been reported [3]. There were no reports of congenital rubella syndrome from 2015 to 2018, but five cases were reported during 2019–2020, and the number of reported cases is still increasing [4].

Therefore, rubella vaccination is important for men and women to control the rubella epidemic, but it is also vital for women who wish to become pregnant to acquire the immunity needed to prevent the infection before pregnancy [2].

In Japan, before the early 1990s, a large-scale epidemic was recognized every five to six years, but from 1995, both male and female infants have been receiving regular vaccination. Since then, no large-scale epidemic has been observed [5]. However, in 2011, a large-scale rubella epidemic broke out in Asia, and consequently, in Japan, sporadic outbreaks caused by the infection in adult men who had been infected abroad and who developed rubella after returning home and their workplace have been reported [6]. Since then, rubella infections have spread rapidly throughout the country [6]. Ninety percent of the reported cases were among adults, and infection in males was approximately 3.5 times more frequent than that in females [6]. Approximately 40% of the reported patients were unvaccinated men born between 2 April 1962 and 1 April 1979 [6]. Since April 2019, the Japanese government has distributed free coupons to men of this age group for undergoing the rubella antibody test. Additionally, the government has administered rubella vaccine free of charge to antibody-negative individuals [6].

Adverse reactions to rubella vaccine include fever [7], rash [7], lymphadenopathy [7], arthralgia [7], hypersensitivity reactions [7], development of immune thrombocytopenia [8], and seizures [8]. However, it is extremely rare for rubella to develop as an adverse reaction to the rubella vaccine. We present the case of a 54-year-old man who developed rubella due to rubella vaccination.

## 2. Case Presentation

A 54-year-old man visited our hospital in Tamba city, Hyogo prefecture, with complaints of back and neck pain for a few days and presented with generalized rash and arthralgia that had persisted for two days before the visit. He had received the first freeze-dried live attenuated rubella vaccine manufactured by Takeda Pharmaceutical (Containing 1000 or more TO-336 STRAIN) 17 days before his visit because he was born in 1965, a generation not routinely vaccinated, as mentioned in the previous section, and had a negative status for rubella antibodies (measles, mumps, and rubella vaccine was not given because his antibody titers for measles and mumps were sufficient). In August 2019, at the time of his vaccination, rubella was prevalent in Hyogo prefecture, mainly in the urban area of the prefecture. From January to the end of August 2019, in Hyogo, the number of reported cases was 47, and the number of reported rubella cases per million people was 8.5 [9]. He then participated in a festival 16 days before his visit and had a history of contact with an unspecified number of people, including spectators from endemic areas. He had a history of hypertension and type 2 diabetes, with a recent HbA1c level of 6.5% and no other history of immunodeficiency. He had been taking calcium antagonists and biguanides for five years. He had no history of traveling abroad.

**Figure 1:**
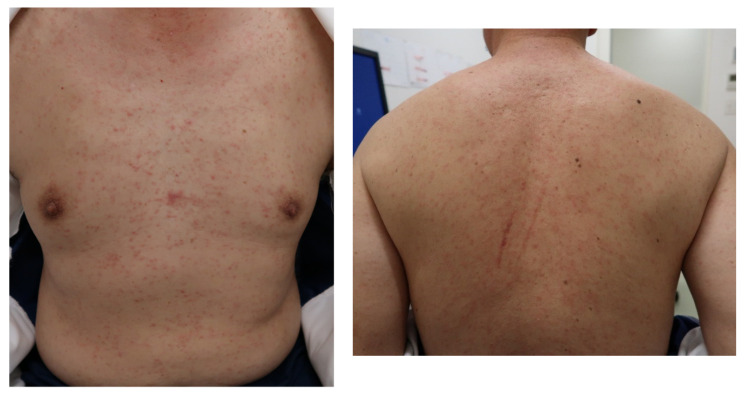
Pink papules throughout the body of the patient.

His vital signs were normal: Body temperature, 35.2 °C; blood pressure, 138/76 mmHg; pulse rate, 87 beats/min; respiratory rate, 12 breaths/min; and peripheral capillary oxygen saturation, 99% at room air. The red spots measuring 1–2 mm were present on his entire body and were itchy. These rashes consisted of pinpoint, pink maculopapules, and did not coalesce (Figure 1). Lymphadenopathy was palpable in the left posterior neck, and no ocular conjunctival hyperemia or oral and palatal enanthem was observed. There were no abnormalities in breath sounds or heart sounds, and no hepatosplenomegaly was observed. At the time of examination, his arthralgia had disappeared.

His blood workup results were as follows: White blood cell count, 6350/µL, with 54.7% neutrophils, 30.1% lymphocytes; red blood cell count, 533 × 10^4^/µL; hemoglobin level, 17.1 g/dL; platelet count, 12.9 × 10^4^/µL; total bilirubin level, 0.7 mg/dL; aspartate aminotransferase level, 56 U/L; alanine aminotransferase level, 94 U/L; serum lactate dehydrogenase level, 237 U/L; γ-glutamyl transferase level, 101 U/L; blood urea nitrogen level, 14.7 mg/dL; creatinine level, 0.76 mg/dL; C-reactive protein level, 0.08 mg/dL. No increase in inflammatory response but mild liver damage was observed. Rapid plasma reagin card agglutination test and Treponema pallidum antibody hemagglutination test for syphilis were both negative, and human immunodeficiency virus antibody test was also negative. He lived in an area free of dengue epidemics and had never traveled to endemic areas. The possibility for syphilis, HIV, and dengue infection was considered low. Based on these laboratory findings, we suggested rubella and other rash-based viral infections. Subsequent additional tests were performed.

The test results are presented in Table 1. The rubella IgM antibody enzyme immunoassay (EIA) titer was 1.48, and the rubella IgG antibody EIA titer was 3.0, revealing a primary rubella infection. Polymerase chain reaction assays for rubella virus detection were performed using blood, pharyngeal swab, and urine specimens, and only the pharyngeal swab yielded a positive result. The genotype was 1a, therefore, it was identified to be a vaccine strain. Based on the results, we made a final diagnosis of rubella as an adverse reaction to rubella vaccination. His symptoms improved after a few days, with no specific treatment administered. The patient was requested to stay at home for three days until the test results were received, after which he returned to work as usual. No subsequent relapse of symptoms and no signs of immunodeficiency were observed.

## 3. Discussion

Herein, we present an extremely rare case of rubella caused by rubella vaccination. The patient was in close contact with people from the rubella endemic area, hence, we had difficulty diagnosing whether he was infected with the vaccine or wild-type strain. Finally, the rubella genotype detected using the pharyngeal swab was 1a. Thus, it was established that the patient was infected with the vaccine strain. The fact that people who develop rubella due to the rubella vaccine have immunodeficiency could not be confirmed in this case or based on previous reports. Therefore, we cannot assert at present that it is necessary to screen for immunodeficiency in people who develop vaccine-related rubella.

In Japan, the Measles–Rubella (MR) combination vaccine has been introduced into the regular vaccination program since 2006, and the vaccine is administered twice at the age of one year and one year before the start of elementary school. From April 2013 to August 2021, among the 20.52 million MR vaccines administered, 453 cases of adverse reactions were reported by medical institutions, of which only one was caused by rubella [10]. There are few reports on the incidence of rubella caused by the rubella vaccine worldwide, and the incidence rate has not been clarified [11].

Rubella virus is a single-stranded enveloped RNA virus belonging to the genus *Rubivirus* of the *Togaviridae* family. It is a virus with no hematologic subtype and has been classified into 13 genotypes through genetic analysis of the E1 protein. In Japan, the genotype 1a strain was mainly detected during 1966–1969. This strain is the source of the current rubella vaccine strain [12]. Therefore, genotype 1a causes rubella as an adverse reaction of the rubella vaccine. Among wild strains, genotype 1j was the main strain in the 2004 epidemic, but since 2011, internationally prevalent genotypes 1E and 2B have emerged and started to spread [12]. At the time of this patient’s examination, the genotypes prevalent in the prefecture were also 1E and 2B [12]. As the patient was infected with genotype 1a, which is a vaccine strain, this infection was deemed to be an adverse reaction due to rubella vaccination.

The vaccine rubella strain is not as highly infectious as the wild-type strain, which can have serious sequelae when adults are infected [13]. Standard precautions and droplet infection precautions are important to prevent wild-type rubella, and people who do not have rubella antibodies should refrain from contact with patients infected with wild-type rubella. It is, however, not necessary to refrain from contact with people infected with vaccine-type rubella.

## 4. Conclusions

This is an extremely rare case of rubella occurring as an adverse reaction after rubella vaccination. The vaccine rubella strain is not as highly infectious as the wild-type strain, which can have serious sequelae when adults are infected. If rubella symptoms appear after rubella vaccination, it must be investigated whether these are due to vaccine-specific adverse reactions, wild-strain rubella onset, or other rash-based viral infections. To curb the rubella epidemic, it is necessary to urgently inoculate people who have not been vaccinated.

## Figures and Tables

**Table 1:** Test results for rash-based viral infections.

Parameter	Antibody Titer	Reference Value	Parameter	Antibody Titer	Reference Value
EBV-VCA-IgM	<10	Less than 0.5	Rubella virus IgM	1.48	Less than 0.8
EBV-VCA-IgG	160	Less than 0.5	Rubella virus IgG	3.0	Less than 2.0
EBNA-IgG	3.1	Less than 0.5	Measles virus IgM	0.06	Less than 0.8
CMV IgM	<0.85	Less than 0.8	Measles virus IgG	35.5	Less than 2.0
CMV IgG	≥250	Less than 2.0	Mumps virus IgM	0.04	Less than 0.8
HSV IgM	0.40	Less than 0.8	Mumps virus IgG	6.2	Less than 2.0
HSV IgG	≥128.0	Less than 2.0	VZV IgM	0.03	Less than 0.8
			VZV IgG	9.9	Less than 2.0

EBV, Epstein–Barr virus; CMV, Cytomegalovirus; HSV, Herpes simplex virus; VZV, Varicella Zoster virus.

## Data Availability

Data sharing is not applicable to this article as no datasets were generated or analyzed during the current study.
